# 

**Published:** 1892-02-27

**Authors:** 


PRICE ONE PENNY.

283, Vol. XI.—February 27th, 1892.

[Registered at the General Post Office as a Newspaper for transmission in the United Kingdom and Abroad.]

WITH EXTRA NURSING SUPPLEMENT
Per Annum, 6s. 6d., post free.
Monthly Parts, 6d.; post free, 8d.
Ditto per Annum, 8s., post free

### THE HOSPITAL

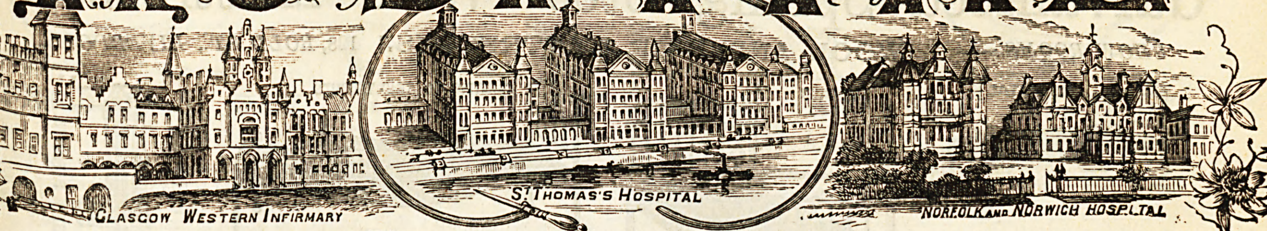

A WEEKLY JOURNAL OF

Science, Medicine, Nursing, and Philanthropy.

### SATURDAY, FEBRUARY 27th, 1892.

**Some Rays of Hope** . . . . . 261
**Passing Topics.**—The Address of the Saturday Fund Chairman . . . . . 262
**The Doctor's Armchair.**—Christianity, Editorship, and Nurses . . . . . 263
**Some Scientific Aspects of Insanity.** XIV.—Secondary Dementia . . . . . 264
**Letters from Far Japan.** By Mrs. Ernest Hart. II.—Hospitals and Medical Education. Part II. . . . . . 264
**Notes and News** . . . . . 266
**General Charities** . . . . . 267
**Annotations.**—The National Drink Bill—Professional Witnesses—Physiologists and Small Holdings . . . . . 268
**Scraps and Gleanings** . . . . . 267
**The Practitioner's Mirror and Retrospect**—
Medicine.—The Symptoms and Treatment of Rhus Poisoning . . . . . 269
Ophthalmology.—Sup urative Keratitis . . . . . 270
Practitioner's Bookshelf.—Yellow Fever, 270; The Journal of Nervous and Mental Disease . . . . . 271
New Drugs, Appliances, and Things Medical.—Pepsalia—Preparations of Pure Beef—Salvine . . . . . 271
**Editor's Letter-Box.**—Vivisection . . . . . 271
**The Hospital Saturday Fund** . . . . . 272
**The Student of Medicine.**—Appointments—Vacancies—Notes from the Schools—Pass List—Athletics.

### EXTRA SUPPLEMENT.—THE NURSING MIRROR.

**En Passant.**—The L.O.S. Examination—The Nurse Dolls—Manchester Nurses—York Home for Nurses—Short Items—The Nurse and the Master—Southend Victoria Hospital—The Hampshire Institute . . . . . cxxvii
**Lectures on Surgical Ward Work and Nursing.** By Alex. Miles, M.D.Edin., F.R.C.S.E. Lecture XLVI. (Conclusion) . . . . . cxxviii
**Appointment** . . . . . cxxix
**For Reading to the Sick.**—Discipline . . . . . cxxix
**On the Nursing of Children.** III.—Operation Cases . cxxx
**Presentations** . . . . . cxxx
**Everybody's Opinion.**—The Nurses' Co-operation—The Sir Alexander Morison Prizes . . . . . cxxxi
**Nurses' Requisites** . . . . . cxxxi
**The Nurses' Bookshelf.**—Swedish Movement—A Community of Women . . . . . cxxxi
**Notes and Queries** . . . . . cxxxi
**Off Duty.**—"The Doctor's Substitute" . . . . . cxxxii

